# Semi-Markov Graph Dynamics

**DOI:** 10.1371/journal.pone.0023370

**Published:** 2011-08-24

**Authors:** Marco Raberto, Fabio Rapallo, Enrico Scalas

**Affiliations:** 1 Dipartimento di Ingegneria Biofisica ed Elettronica, Università degli Studi di Genova, Genova, Italy; 2 Dipartimento di Scienze e Tecnologie Avanzate, Università del Piemonte Orientale “Amedeo Avogadro”, Alessandria, Italy; 3 Basque Center for Applied Mathematics, Bizkaia Technology Park, Derio, Spain; University of Maribor, Slovenia

## Abstract

In this paper, we outline a model of graph (or network) dynamics based on two ingredients. The first ingredient is a Markov chain on the space of possible graphs. The second ingredient is a semi-Markov counting process of renewal type. The model consists in subordinating the Markov chain to the semi-Markov counting process. In simple words, this means that the chain transitions occur at random time instants called epochs. The model is quite rich and its possible connections with algebraic geometry are briefly discussed. Moreover, for the sake of simplicity, we focus on the space of undirected graphs with a fixed number of nodes. However, in an example, we present an interbank market model where it is meaningful to use directed graphs or even weighted graphs.

## Introduction

The publication of *Collective dynamics of ‘small world’ networks* by Watts and Strogatz [1] gave origin to a plethora of papers on network structure and dynamics. The history of this scientific fashion is well summarized by Rick Durrett [2]:

The theory of random graphs began in the late 1950s in several papers by Erdös and Rényi. In the late twentieth century, the notion of six degrees of separation, meaning that any two people on the planet can be connected by a short chain of people who know each other, inspired Strogatz and Watts [1] to define the small world random graph in which each side is connected to *k* close neighbors, but also has long-range connections. At about the same time, it was observed in human social and sexual networks and on the Internet that the number of neighbors of an individual or computer has a power law distribution. This inspired Barabási and Albert [3] to define the preferential attachment model, which has these properties. These two papers have led to an explosion of research. While this literature is extensive, many of the papers are based on simulations and nonrigorous arguments.

Incidentally, the results of Watts and Strogatz were inspired by the empirical and theoretical work by Milgram [4] and Granovetter [5] back in the 1960s and 1970s; similarly, the preferential attachment model by Barabási and Albert is closely related to the famous 1925 paper by Yule [6] as well as to a celebrated work by Herbert Simon published in 1955 [7] (see also chapters 8 and 9 in reference [8] for a recent analysis on Simon's results). This body of literature is partially reviewed in Durrett's book [2] as well as in a popular science book written by Barabási [9].

It might be interesting to understand why this scientific fashion was born and how. On this respect, we can quote Wikipedia's article (as retrieved on 4 May 2011) on Milgram's experiment in popular culture [10]:

Social networks pervade popular culture in the United States and elsewhere. In particular, the notion of six degrees has become part of the collective consciousness. Social networking websites such as Friendster, MySpace, XING, Orkut, Cyworld, Bebo, Facebook and others have greatly increased the connectivity of the online space through the application of social networking concepts. The “ Six Degrees” Facebook application calculates the number of steps between any two members. […]

In other words, the social character of human beings combined with the hyper-simplification (trivialization) of some results promoted by leading science journals might have triggered interest in social networkology also outside scientific circles. Moreover, the emergence of social networks in the Internet has indeed made some tools developed by networkologists profitable. However, a deeper analysis by sociologists and historians of science will be necessary to falsify or corroborate such hypotheses.

In this paper, we pay our tribute to this fashion, but we slightly depart from the bulk of literature on social network dynamics. First of all we consider time evolution also in *continuous time* and not only in discrete time. As the reader will see, this will be enough to give rise to interesting non stationarities as well as to non-trivial ergodic behavior. Moreover, to begin with a simple situation, we will be concerned with *undirected graphs* whose number of nodes *M* does not change in time. These restrictions can be easily overcome and, indeed, in the following, an example with *directed graphs* will be presented. The dynamic variable will be the *topology* of the graph. This approach is motivated by the following considerations. Social networks are intrinsically volatile. You can be in contact with someone for a finite time (at a meeting, during a phone call, etc.), but never meet this person again in the future. This interaction may or may not have effects on your future actions. If memory is not a major issue, the new configuration of the graph will only depend on the previous configuration. Memory *is* indeed an issue, but again, to simplify the analysis, we will consider a semi-Markov dynamics on the state space of all the possible graphs with *M* nodes. It is already quite rich. Incidentally, notice that all finite-memory processes in discrete time can be re-written as Markov chains.

The dynamics will be defined by a Markov chain subordinated to a generic counting process. Similar models have been around for many years. They were (and are) commonly used in engineering and decision analysis and, on this point, the interested reader can consult the monograph by Howard [11].

In this framework, it is often assumed that the waiting times between consecutive events do follow the exponential distribution, so that the corresponding counting process is Poisson. Indeed, many counting processes with non-stationary and non-independent increments converge to the Poisson process after a transient. If these counting processes are of renewal type, i.e. inter-arrival times 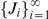 are independent and identically distributed (iid) random variables, it is sufficient to assume that the expected value of these inter-arrival times is finite. However, recently, it has been shown that heavy-tailed distributed interarrival times (for which 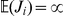) play an important role in human dynamics [12]–[14]. After defining the process in the Methods Section, we will present two introductory examples and a detailed model of an interbank market in the Results Section.

## Methods

This section begins with the definition of the two basic ingredients of our model, namely

1. a *discrete-time Markov chain* on the finite set of 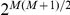 undirected graphs with 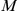 vertices (nodes), and

2. a counting process 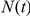 for the point process corresponding to a renewal process.

The rest of the section is devoted to the definition of the basic model class.

### Ingredient 1: a Markov chain on graphs

Consider an undirected graph 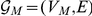 where 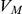 represents a set of 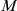 vertices (nodes) and 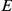 the corresponding set of edges. Any such undirected graph can be represented by a symmetric 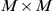 adjacency matrix 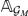, or simply 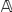, with entries 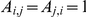 if vertices 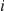 and 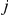 are connected by an edge and 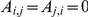 otherwise. Note that algebraic graph theory using linear algebra leads to many interesting results relating spectral properties of adjacency matrixes to the properties of the corresponding graphs [15], [16]. For instance, the matrix
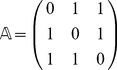
(1)


corresponds to a graph where there are no self-connections and each vertex is connected to the other two vertices. As mentioned above, for a given value of 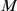 there are 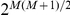 possible graphs. To see that, it is sufficient to observe that the 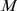 diagonal entries can assume either value 1 or value 0 and the same is true for the 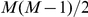 upper diagonal entries. Now, denote by 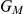 the set of 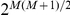 undirected graphs with 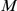 nodes. Consider a sequence of random variables 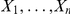 assuming values in 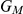. This becomes our *state space*, and the set of 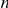 random variables is a finite stochastic process. Its full characterization is in term of all finite dimensional distributions of the following kind (for 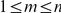) [17]

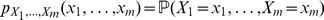
(2)


where 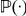 denotes the probability of an event with the values 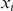 running on all the possible graphs 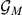 of 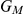. The finite dimensional distributions defined in equation (2) obey the two compatibility conditions of Kolmogorov [17], namely a symmetry condition
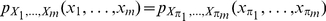
(3)


for any permutation 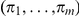 of the 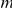 random variables (this is a direct consequence of the symmetry property for the intersection of events) and a second condition
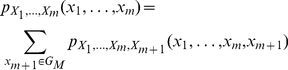
(4)


as a direct consequence of total probability.

Among all possible stochastic processes on 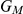, we will consider *homogeneous Markov chains*. They are fully characterized by the *initial probability*

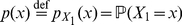
(5)


and by the transition probability
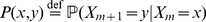
(6)


that does not depend on the specific value of 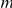 (hence the adjective homogeneous). Note that it is convenient to consider the initial probability as a row vector with 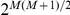 entries with the property that
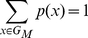
(7)


and the transition probability as a 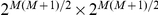 matrix, also called *stochastic matrix* with the property that
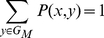
(8)


For a homogeneous Markov chain, the finite dimensional distributions are given by
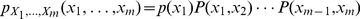
(9)


It is a well known fact that the finite dimensional distributions in equation (9) do satisfy Kolmogorov's conditions (3) and (4). Kolmogorov's extension theorem then implies the existence of Markov chains [17]. Marginalization of equation (9) leads to a formula for 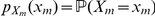, this is given by
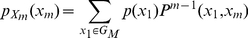
(10)


where 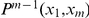 is the entry 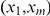 of the 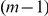-th power of the stochastic matrix. Note that, from equation (10) and homogeneity one can prove the *Markov semi-group property*

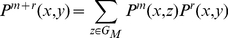
(11)


Starting from the basic Markov process with the set of graphs as space state, we can also consider other auxiliary processes. Just to mention few among them, we recall:

- the process counting the number of edges (i.e., the sum of the adjacency matrix 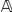);
- the process recording the degree of the graph (i.e., the marginal total of the adjacency matrix 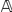);
- the process which measures the cardinality of the strongly connected components of the graph.

Notice that the function of a Markov chain is not a Markov chain in general, and therefore the study of such processes is not trivial.

Under a more combinatorial approach, one can consider also the process recording the permanent of the adjacency matrix 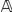. We recall that the permanent of the matrix 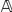 is given by
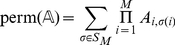
(12)


where 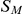 is the symmetric group on the set 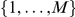. The permanent differs from the best known determinant only in the signs of the permutations. In fact,
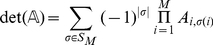
(13)


where 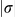 is the parity of the permutation 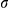. Notice that the permanent is in general harder to compute than the determinant, as Gaussian elimination cannot be used. However, the permanent is more appropriate to study the structure of the graphs. It is known, see for instance [16], that the permanent of the adjacency matrix counts the number of the bijective functions 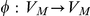. The bijective functions 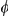 are known in this context as perfect matchings, i.e., the rearrangements of the vertices consistent with the edges of the graph. The relations between permanent and perfect matchings are especially studied in the case of bipartite graphs, see [18] for a review of some classical results.

Moreover, we can approach the problem also from the point of view of *symbolic computation*, and we introduce the *permanent polynomial*, defined for each adjacency matrix as follows. Let 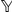 be an 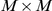 matrix of variables 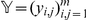. The permanent polynomial is the polynomial
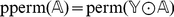
(14)


where 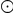 denotes the element-wise product. For example, the polynomial determinant of the adjacency matrix
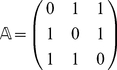
(15)


introduced above is
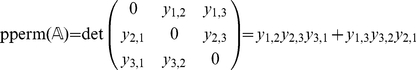
(16)


The permanent polynomial in Equation (14) is a homogeneous polynomial with degree 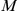 and it has as many terms as the permanent of 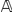, all monomials are pure (i.e., with unitary coefficient) and each transition of the Markov chain from the adjacency matrix 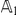 to the matrix 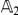 induces a polynomial 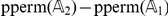.

Finally, it is also interesting to consider conditional graphs. With this term we refer to processes on a subset of the whole family of graphs 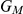. For instance we may require to move only between graphs with a fixed degree, i.e., between adjacency matrices with fixed row (and column) totals. In such a case, also the construction of a connected Markov chain in discrete time is an open problem, recently approached through algebraic and combinatorial techniques based on the notion of Markov basis, see [19]–[21]. This research topic, named *Algebraic Statistics* for contingency tables, seems to be promising when applied to adjacency matrices of graphs.

### Ingredient 2: a semi-Markov counting process

Let 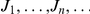 be a sequence of positive independent and iid random variables interpreted as sojourn times between events in a point process. They are a *renewal process*. Let
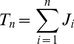
(17)


be the *epoch* (instant) of the 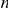-th event. Then, the process 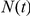 counting the events occurred up to time 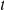 is defined by
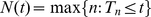
(18)


A well-known (and well-studied) counting process is the Poisson process. If 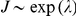, one can prove that
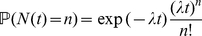
(19)


The proof leading to the exponential distribution of sojourn times to the Poisson distribution of the counting process is rather straightforward. First of all one notices that the event 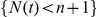 is given by the union of two disjoint events
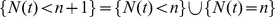
(20)


therefore, one has
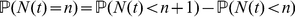
(21)


but, by definition, the event 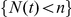 coincides with the event 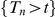. Therefore, from equation (21), one derives that
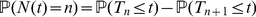
(22)


The thesis follows from equation (17). The cumulative distribution function of 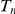 is the 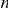-fold convolution of an exponential distribution, leading to the *Erlang distribution*

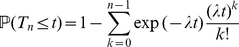
(23)


and, by virtue of equation (22), the difference 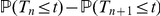 gives the Poisson distribution of equation (19). Incidentally, it can be proved that 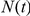 has stationary and independent increments.

One can also start from the Poisson process and then show that the sojourn times are iid random variables. The Poisson process can be defined as a non-negative integer-valued stochastic process 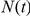 with 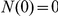 and with stationary and independent increments (i.e. a Lévy process; it must also be stochastically continuous, that is it must be true that for all 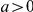, and for all 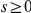 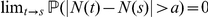) such that its increment 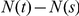 with 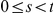 has the following distribution for 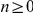


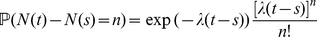
(24)


Based on the definition of the process, it is possible to build any of its finite-dimensional distributions using the increment distribution. For instance 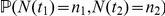 with 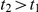 is given by
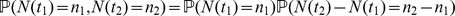


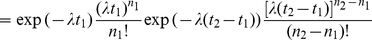
(25)


Every Lévy process, including the Poisson process is Markovian and has the so-called *strong Markov property* roughly meaning that the Markov property is true not only for deterministic times, but also for random stopping times. Using this property, it is possible to prove that the sojourn times are iid. For 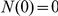, let 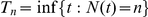 be the 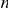-th epoch of the Poisson process (the time at which the 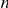-th jump takes place) and let 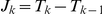 be the 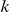-th sojourn time (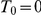). For what concerns the identical distribution of sojourn times, one has that
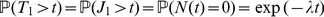
(26)


and for a generic sojourn time 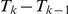, one finds
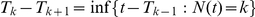


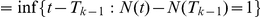


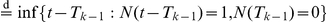


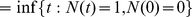
(27)


where 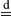 denotes equality in distribution and the equalities are direct consequences of the properties defining the Poisson process. The chain of equalities means that every sojourn time has the same distribution of 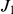 whose survival function is given in equation (26). As mentioned above, the independence of sojourn times is due to the strong Markov property. As a final remark, in this digression on the Poisson process, it is important to notice that one has that its *renewal function* 
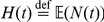
 is given by
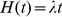
(28)


i.e. the renewal function of the Poisson process linearly grows with time, whereas its renewal density 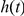 defined as
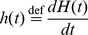
(29)


is constant:
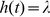
(30)


Here, for the sake of simplicity, we shall only consider renewal processes and the related counting processes (see equations (17) and (18)). When sojourn times are non-exponentially distributed, the corresponding counting process 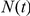 is no longer Lévy and Markovian, but it belongs to the class of *semi-Markov* processes further characterized in the next section [22]–[25]. If 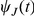 denotes the probability density function of sojourn times and 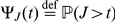 is the corresponding survival function, it is possible to prove the *first renewal equation*

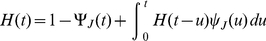
(31)


as well as the *second renewal equation*

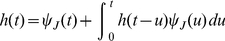
(32)


The second renewal equation is an immediate consequence of the first one, based on the definition of the renewal density 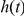 and on the fact that 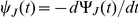. The first renewal equation can be obtained from equation (22) which is valid in general and not only for exponential waiting times. One has the following chain of equalities
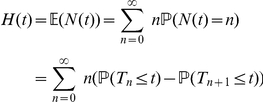


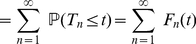
(33)


where 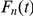 is the cumulative distribution function of the random variable 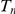, a sum of iid. positive random variables. Let 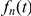 represent the corresponding density function. By taking the Laplace transform of equation (33) and using the fact that
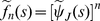
(34)


one eventually gets
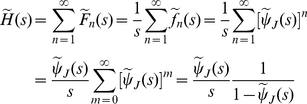
(35)


or (as 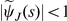 for 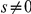)
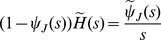
(36)


the inversion of equation (36) yields the first renewal equation (31).

If the sojourn times have a finite first moment (i.e. 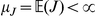), one has a *strong law of large numbers* for renewal processes
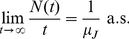
(37)


and as a consequence of this result, one can prove the so-called *elementary renewal theorem*

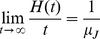
(38)


The intuitive meaning of these theorems is as follows: if a renewal process is observed a long time after its inception, it is impossible to distinguish it from a Poisson process. As mentioned in the Introduction, the elementary renewal theorem can explain the ubiquity of the Poisson process. After a transient period, renewal processes with finite first moment behave as the Poisson process. However, there is a class of renewal processes for which the condition 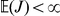 is not fulfilled. These processes never behave as the Poisson process. A prototypical example is given by the renewal process of Mittag-Leffler type introduced by one of us together with F. Mainardi and R. Gorenflo back in 2004 [26], [27]. A detailed description of this process will be given in one of the examples below.

### Putting the ingredients together

Let 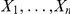 represent a (finite) Markov chain on the state space 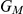, we now introduce the process 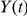 defined as follows
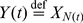
(39)


that is the Markov chain 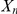 is *subordinated* to a counting process 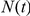 coming from a renewal process as discussed in the previous subsection, with 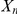 independent of 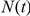. In other words, 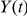 coincides with the Markov chain, but the number of transitions up to time 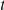 is a random variable ruled by the probability law of 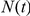 and the sojourn times in each state follow the law characterized by the probability density function 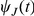, or, more generally, by the survival function 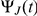.

As already discussed, such a process belongs to the class of semi-Markov processes [22]–[25], [28], i.e. for any 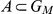 and 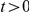 we do have
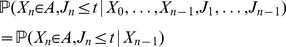
(40)


and, if the state 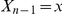 is fixed at time 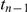, the probability on the right-hand side will be independent of 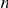. Indeed, by definition, given the independence between the Markov chain and the counting process, one can write
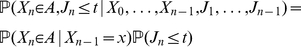
(41)



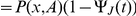
41)

where
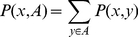
(42)


Equation (41) is a particular case of (40).

It is possible to introduce a slight complication and still preserve the semi-Markov property. One can imagine that the sojourn time in each state is a function of the state itself. In this case 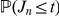 is no longer independent of the state of the random variable 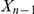 and equation (41) is replaced by
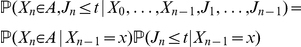


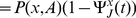
(43)


where 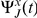 denotes the state-dependent survival function. However, in this case, the random variable 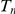 is still the sum of independent random variables, but they are no-longer identically distributed, and the analysis of the previous section has to be modified in order to take this fact into proper account.

## Results

In order to show the behavior of the stochastic processes described in the previous sections we have simulated the distribution of two stopping times in two different situations. The simulations have been written in R, see [29] and the source files are available as Supporting Information S1 and S2 Notice that some specific packages for the analysis of graph structures are available, see for instance [30]. However, we have used only the R-base commands, so that our examples can be analyzed easily without any additional package.

The examples in the first two subsections are designed to introduce the reader to the simulation algorithms in a framework as simple as possible. An extended example about a model of interbank market will be discussed in the last subsection.

In our examples we use the Mittag-Leffler distribution for the sojourn times. We recall that the Mittag-Leffler distribution has survival function given by
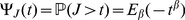
(44)


where 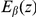 is the one-parameter Mittag-Leffler function defined as
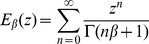
(45)


for 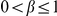. There are two strong reasons for this choice. The first one is that many analytical results are available on the Mittag-Leffler renewal process a.k.a. fractional Poisson process [26], [27], [31]–[34]. The second reason is that the Mittag-Leffler distribution is the repeated-thinning limit of heavy-tailed sojourn-time distributions with algebraically decaying tails with exponent 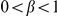 [27]. For 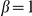, the exponential distribution is recovered from (44).

### First example

In this example we consider graphs without self-loops. Let us consider a fixed number 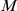 of vertices and define a process as follows:

- At time 0, there are no edges in the graph;
- At each time, we choose an edge 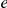 with uniform distribution on the 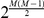 edges. If 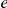 belongs to the graph we remove it; if 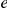 does not belong to the graph we add it;
- The stopping time is defined as the first time for which a triangle appears in the graph.

To simulate the distribution of the stopping times we have used 10000 replications. As the Mittag-Leffler distribution is heavy-tailed, the density plot and the empirical distribution function plot are not informative. Thus, we have reported the box-plot, to highlight the influence of the outliers.

**Figure 1:**
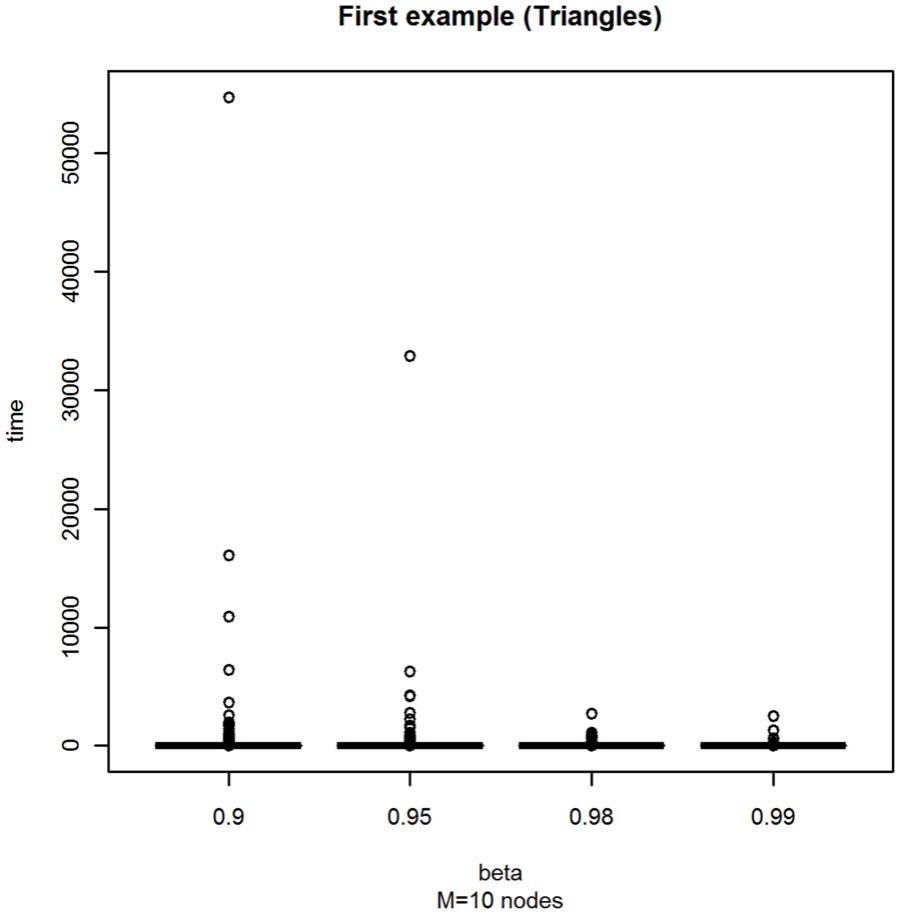
Box-plot of the distribution of the stopping times with varying *β* for Example A.

With a first experiment, we have studied the influence of the *β* parameter. In a graph with 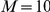 nodes, we have considered the sojourn times with a Mittag-Leffler distribution with different *β* parameter, namely 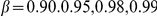. The box-plots are displayed in Figure 1, and some numerical indices are in Table 1.

**Table 1:** Summary statistics for Example A with varying *β*.

	Min	1*^st^* Qu.	Median	Mean	3*^rd^* Qu.	Max
*β* = 0.9	0.363	8.010	12.750	31.460	20.260	54750.000
*β* = 0.95	0.325	7.545	11.440	20.860	16.890	32920.000
*β* = 0.98	0.261	7.296	10.860	12.950	15.050	2704.000
*β* = 0.99	0.537	7.219	10.630	12.340	14.670	2487.000

Our results show that:

- the outliers are highly influenced from the value of *β*. This holds, with a less strong evidence, also for the quartiles 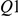 and 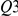;
- the median is near constant, while the mean is affected by the presence of outliers.

**Figure 2:**
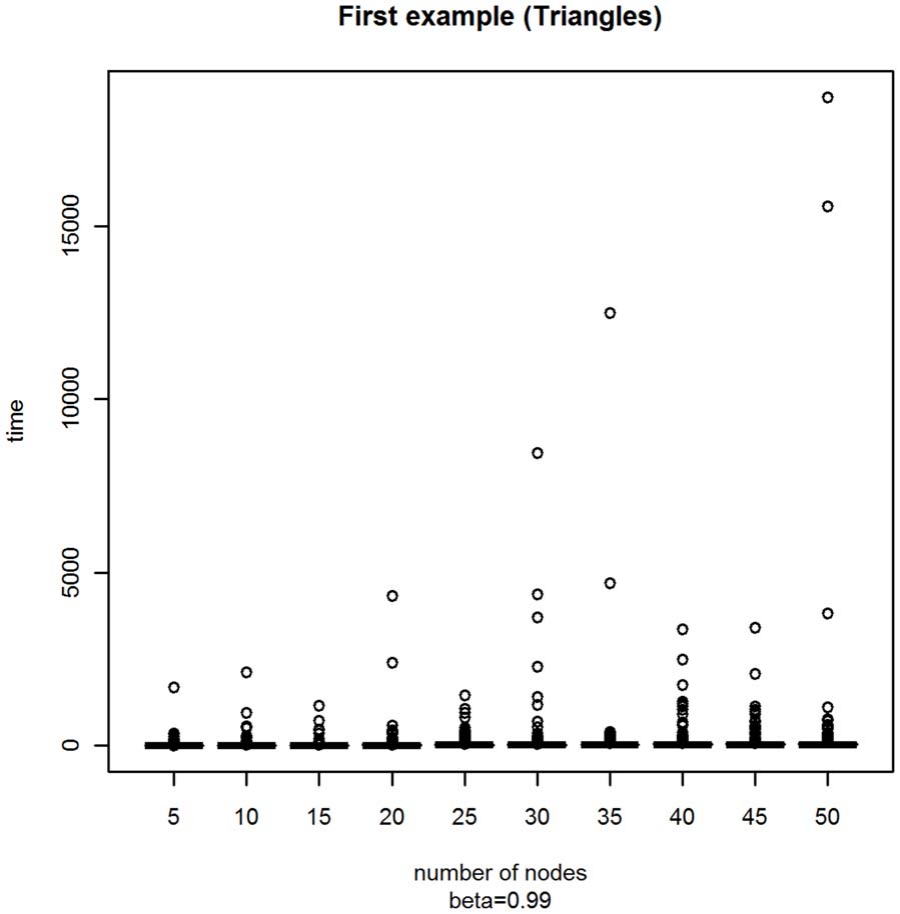
Box-plot of the distribution of the stopping times with varying *M* for Example A.

**Figure 3:**
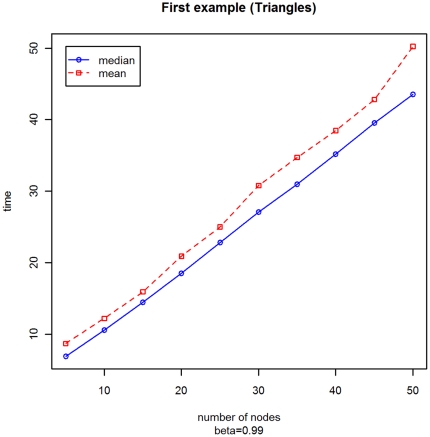
Mean and median of the distribution of the stopping times with varying *M* for Example A.

With a second experiment, we have considered a fixed parameter *β* = 0.99, but a variable number of vertices *M* ranging from 5 to 50 by 5. In Figures 2 and 3 we present the box-plots of the stopping time distribution and the trends of the mean and the median. From these graphs we can notice that:

- the presence of outliers is more relevant in the graph with a large number of nodes;
- the mean and the median are roughly linear, but the trend of the median looks more stable.

### Second example

Let us consider a population with individuals 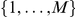 and suppose that the person labelled 1 has to share some information. At a first random time, he chooses another individual with random uniform probability and shares the information with him. At a second random time, one person who has the information chooses an individual among the other 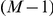 and shares again the information. Note that each individual shares the information with another one, no matter if the latter has already or not the information. At each time, we denote by 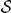 the subset of persons having the information. In terms of graphs, the process is then defined as follows:

- At time 0, there are no edges in the graph and 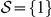;
- At each time, we choose a vertex 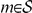 and we choose an edge among 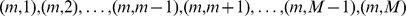 with random uniform distribution. If the chosen edge is already in the graph we do nothing; otherwise, we add the chosen edge to the graph and we add the appropriate vertex to 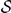;
- The stopping time is defined as the first time for which the whole graph is connected.

**Figure 4:**
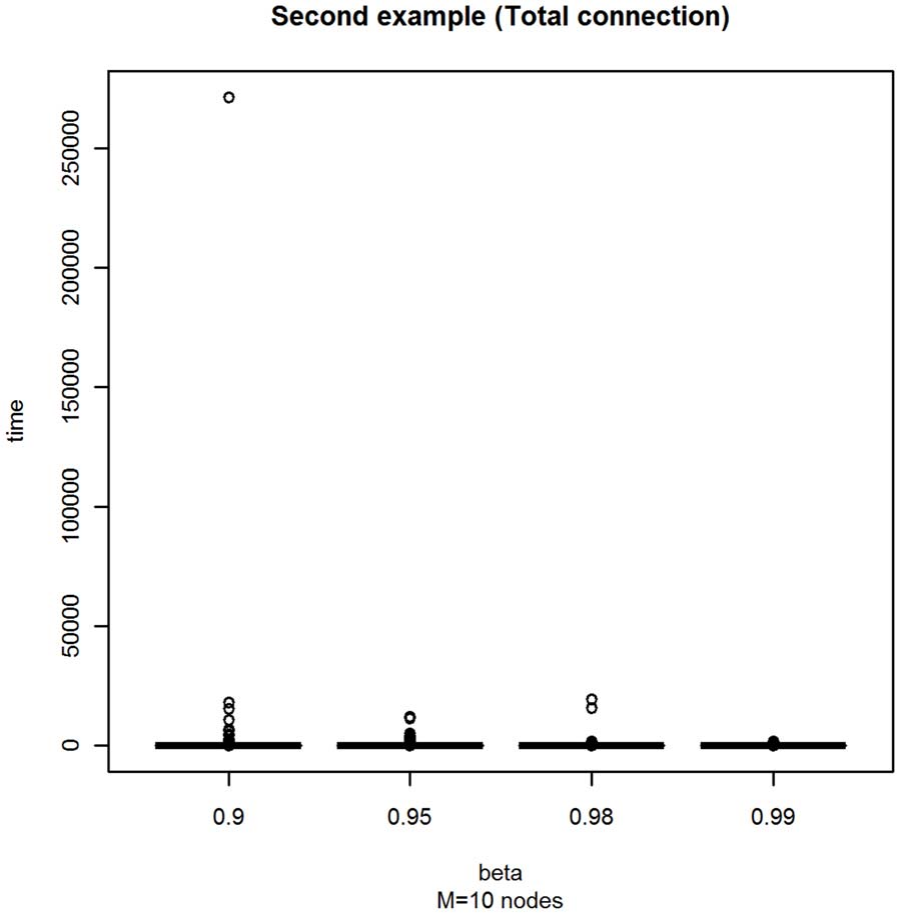
Box-plot of the distribution of the stopping times with varying *β* for Example B.

The experimental settings for this example are the same as for Example A. With a fixed number of vertices 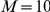 and varying *β* as above, we obtain the box-plots in Figure 4, and the numerical summary in Table 2. From these results we can see that the outliers are highly influenced from the value of *β*, while the variation of the quantiles 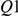 and 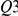 is much lower. Also in this example, the mean is affected by the presence of outliers.

**Table 2:** Summary statistics for Example B with varying *β*.

	Min	1*^st^* Qu.	Median	Mean	3*^rd^* Qu.	Max
*β* = 0.9	3.786	21.540	32.070	88.110	49.380	271500.000
*β* = 0.95	3.393	19.410	27.050	41.550	38.260	12140.000
*β* = 0.98	3.565	18.230	24.980	33.530	33.970	19600.000
*β* = 0.99	4.738	17.690	23.940	27.160	32.310	1701.000

**Figure 5:**
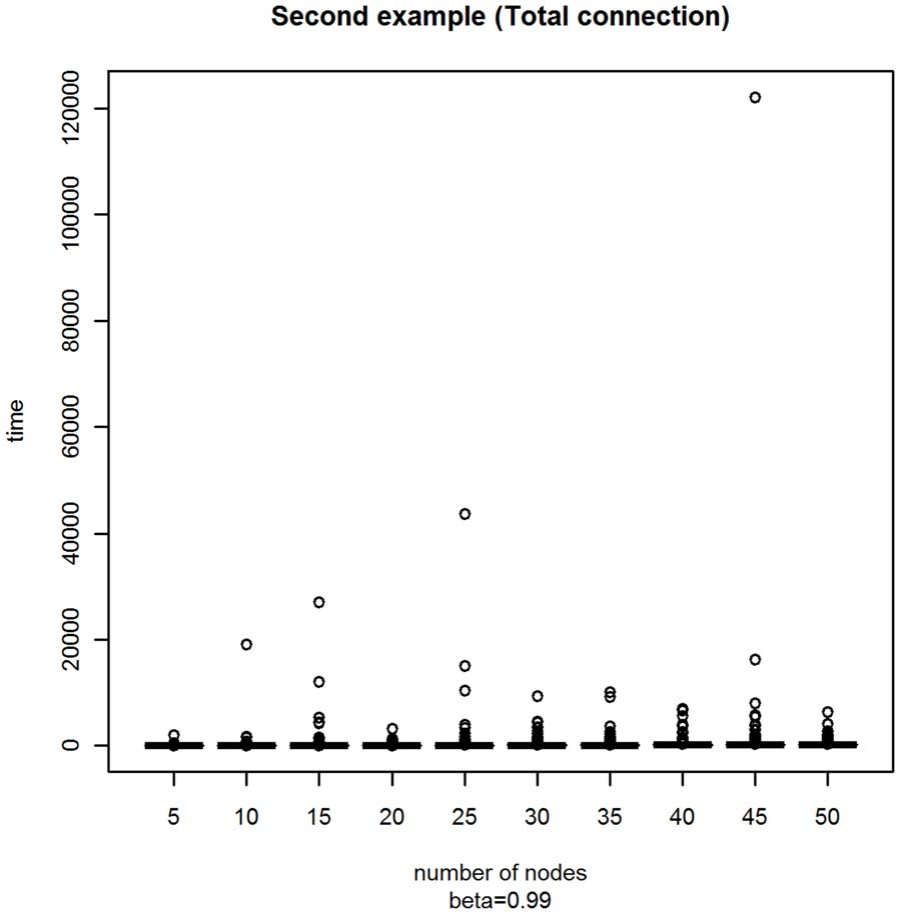
Box-plot of the distribution of the stopping times with varying *M* for Example B.

**Figure 6:**
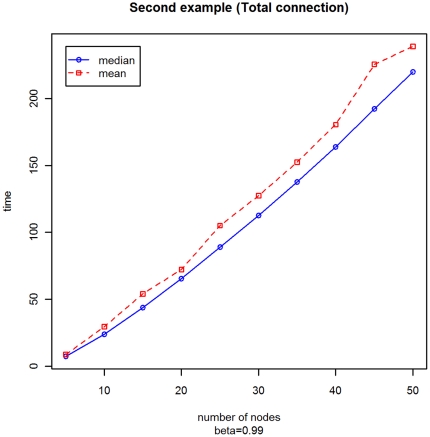
Mean and median of the distribution of the stopping times with varying *M* for Example B.

With the second experiment with a variable number of vertices *M* ranging from 5 to 50 by 5, we obtain the plots displayed in Figures 5 and 6. The conclusions are the same as in the previous example.

### Extended example. An interbank market

In this subsection we present a simple model for interbank markets. It serves the purpose of illustrating the modelling potential of the ideas presented above.

This example deals with an interbank market receiving loan requests from the corporate sector at random times. For the sake of readability, in this subsection we will use the symbol 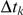 instead of 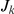 for the 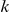-th inter-arrival duration and we will denote the epochs at which loan requests are made with the symbol 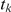 instead of 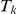. In this more realistic example, we will briefly discuss the difficulties that must be faced when one desires to go beyond a mere phenomenological description of reality.

We consider an interbank market characterized by 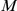 banks that demand and supply liquidity at a given interest rate 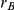. Each bank 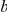 is described at any time by its balance sheet, as outlined in Table 3. The market is decentralized and banks exchange liquidity by means of pairwise interactions. Banks lend money also to the corporate sector at the constant rate 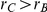 and all corporate and interbank loans are to be repayed after *T* units of time. We stipulate that the loan requests from the corporate sector to the banking system are the events triggering the interbank market and we model these events as a Poisson process of parameter 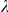. In particular, we state that, at exponentially distributed intervals of time 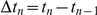, a loan request of constant amount 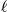 is submitted from the corporate sector to a bank chosen at random with uniform distribution among the *M* banks. As in the previous examples, in principle, the Poisson process can be replaced by any suitable counting process. Let us denote the chosen bank with the index 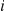 and the time at which the loan is requested as 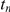. If 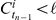, the chosen bank is short of liquidity to grant the entire amount of the loan. Given the interest rate spread between 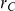 and 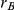, the profit-seeking bank enters the interbank market in order to borrow at the rate 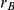 the amount 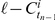 necessary to grant the entire loan. In the interbank market, a new bank is then chosen at random with uniform distribution among the remaining 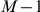 banks. Let us denote with 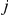 the new chosen bank. If bank 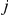 has not enough liquidity to lend the requested amount, i.e., if 
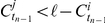
, then a new bank 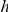 is again chosen at random among the remaining 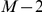 ones to provide the residual liquidity, and so on. This process in the interbank market continues till the liquidity amount 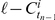 necessary to bank 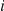 is collected.

**Table 3:** Balance sheet entries of bank b at time *t_n_*.

Assets	Liabilities
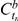 : liquidity	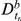 : total (households' and firms') deposits
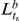 : loans to the corporate sector	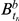 : debt with other banks
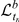 : loans to other banks	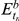 : equity (net worth)

Finally, as soon as the loan 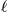 is provided to the corporate sector, we stipulate that the deposits as well as the liquidity of any bank 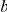, being 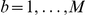, is increased by the amount 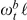, where 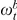 are random numbers constrained by 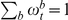. The rationale behind this choice is that a loan, when it is taken and spent, creates a deposit in the bank account of the agent to whom the payment is made; for instance, when the corporate sector gets a loan to pay wages to workers or to pay investments to capital goods producers, then the deposits at the *M* banks of the agents receiving the money are increased by a fraction of the borrowed amount 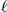. We assume that these deposits are randomly distributed among the *M* banks.

To give a clearer idea on how the balance sheets of banks evolve after an event in the interbank market, let us consider an example where at time 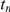 the corporate sector requests a loan 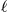 to the randomly selected bank 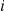, which, being short of liquidity (i.e. 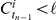), needs to enter into interbank market where it borrows a loan of amount 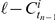 from the randomly selected bank 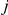. We suppose here 
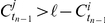
, therefore no other lending banks enter the interbank market. According to the model outlined above, at the end of the interbank market session, the balance sheets of bank 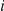 and of bank 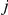 change as outlined in Table 4.

**Table 4:** Dynamics of balance sheet entries of bank 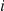 (lender to the corporate sector and borrower in the interbank market) and bank 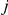 (lender in the interbank market) at time *t* ***_n_*** when both the corporate loan 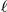 and the related interbank loan 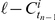 are granted.

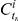	=	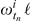	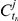	=	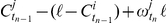
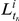	=	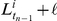	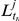	=	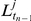
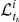	=	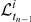	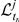	=	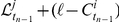
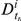	=	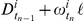	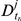	=	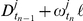
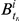	=	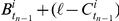	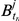	=	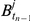
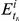	=	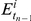	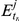	=	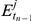

Once the assets and the debt liabilities entries of any bank are updated following the lending activity to the corporate sector and the interbank market outcomes, the equity is then updated as residual according to the usual accounting equation:
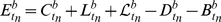
(46)


It is worth noting that, as reported in Table 4, the equity of both bank 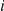 and 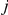 does not change from 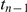 to 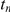. This result is obtained by computing the new equity levels at time 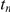 using (46) but should not be a surprise given that lending and borrowing clearly change the balance sheet entries of banks but not their net worth at the time the loan is granted or received. Indeed, the net worth of the lending banks is increased by the interest revenues when the corporate loan as well as the interbank loan is repaid together with the interest amounts. In particular, equity of bank 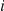 is increased by 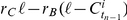, while equity of bank 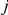 is increased by 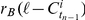. Table 5 shows how balance sheet entries change at time 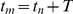 when the two loans are paid back. It is worth noting again that the equity dynamics is consistent with the dynamics of other balance sheet entries, according to (46). Finally, as granting a bank loan to the corporate sector increases private deposits at banks, also the opposite holds when a loan is paid back. The repayment of the loan 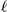 together with interests 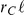 corresponds to a reduction of private deposits, as well as of the related banks' liquidity, of the same amount. As in the previous case, we assume that the reduction 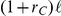 is uniformly and randomly distributed among the *M* banks with weights 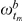, where 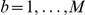.

**Table 5:** Dynamics of balance sheet entries of bank 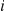 (lender to the corporate sector and borrower in the interbank market) and bank 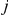 (lender in the interbank market) at time 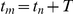 when both the corporate loan 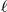 and the related interbank loan 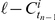 are paid back.

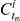	=	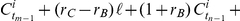	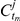	=	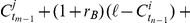
		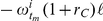			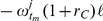
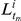	=	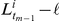	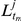	=	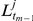
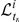	=	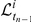	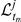	=	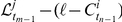
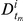	=	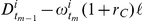	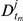	=	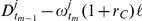
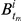	=	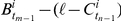	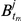	=	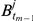
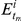	=	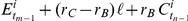	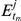	=	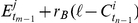

We can then define a 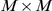 adjacency matrix 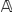 representing the graph associated to the interbank market, where the nodes of the graph correspond the 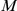 banks and the edges to the lending and borrowing relationships among banks. Differently from the previous discussion and examples, here, it is meaningful to consider directed graphs and therefore the matrix can be asymmetric. In particular, if bank 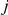 is lending money to bank 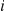, we set 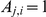, but we may have 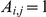 or 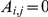, depending if bank 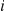 is lending or not money to bank 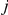. The situation where both 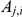 and 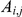 are set to 1 is not contradictory but it means that two loans have been granted in the two opposite directions, i.e. from bank 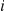 to bank 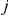 and from bank 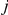 to bank 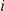, at different times. In fact, let us suppose that at time 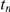, as stated in the example, bank 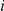 borrows money from bank 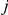 so that 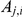 is set to 1, while 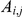 is still zero. The loan will be repaid at time 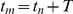, but it may happen that that at any time 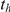, being 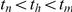, bank 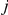 has been randomly chosen as the corporate sector lender, and, being short of liquidity, bank 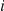 is chosen to provide the necessary liquidity in the interbank market. Bank 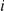 is likely to have 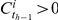 and so able to lend to bank 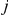. The reason is that bank 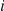 ended period 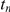 with a positive liquidity, i.e., 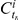  =  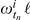, see Table 4 and the related discussion; moreover, we cannot exclude that a loan granted by bank 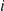 in the past has been repaid at any time between 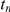 and 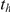. Therefore, if the conditions above are all verified, it will happen that both 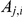 and 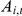 are equal to 1 at any time t in between 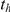 and 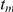. The overall result can be interpreted as a net lending between one bank to the other, the direction depends on the amounts of money involved, but the two loan cannot be cancelled out because they have been granted and they will expire at different times.

The time evolution of the adjacency matrix depends on the evolution of events in the interbank market. In particular, when the first loan from bank 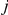 to bank 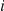 is paid back, 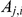 is again set to 0, provided that no new loans have been granted by bank 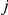 to bank 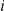 in the meantime, if this happens the value of 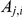 remains at 1 till there are debts of bank 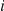 to bank 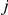. If this is required by the particular application, it is even possible to consider *weighted graphs* where the entry 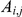 contains the value of the loan from bank 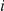 to bank 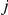.

The dynamics in the interbank market can then be represented as a Markov chain on graphs subordinated to the Poisson process representing the random events of loan requests to the banking system by the corporate sector. It is worth noting that the Markov process and the Poisson process are independent here, however, the transition probabilities of the Markov process are not fixed *ex ante* but depends on the endogenous evolution of the balance sheets of banks. Therefore, here, the Markov process is *not homogeneous*.

## Discussion

We have discussed a model of graph dynamics based on two ingredients. The first ingredient is a Markov chain on the space of possible graphs. The second ingredient is a semi-Markov counting process of renewal type. The model consists in subordinating the Markov chain to the semi-Markov counting process. In simple words, this means that the chain transitions occur at random time instants called epochs. This model takes into account the fact that social interactions are intrinsically volatile and not permanent.

Note that state dependent subordination (see equation (43)) gives rise to very interesting dynamics from the ergodicity viewpoint [35]. In order to illustrate this fact, let us consider a simple two-state aperiodic and irreducible Markov chain with the following transition probability matrix:
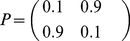
(47)


In this case, the invariant measure is uniform and it is given by
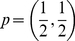
(48)


meaning that the probability of finding each state at equilibrium is 1/2. Now, let us call *A* the first state and *B* the second state. Let the sojourn time in *A* be exponentially distributed with parameter 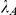 and the sojourn time in *B* still exponentially distributed with parameter 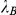. If a single realization of this process is considered, the average time of permanence in state *A* will be given by 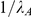 and the average time of permanence in *B* will be given by 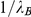. Therefore, if 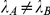, then the ratio of average sojourn times will be different from 1. In other words, for this simple model, the fraction of sojourn times is not equal to the fraction of the ensemble measure: a signal of non-ergodicity.

Finally, with reference to the examples discussed above, this kind of modeling can be used for risk evaluation. Given a loss function, a function that gives the losses when adverse events take place, the risk function is defined as the expected value of the loss function. With our approach, one can derive the probability of the adverse events as a function of time and use this measure to evaluate the risk function. To be more specific, assume that the failure of a bank implies the payment of deposit insurance up to a certain limit. Then the loss function can be defined as the capped sum of deposits and risk is the product of this loss function by the bank failure probability at a certain time instant. The latter example may become relevant for macroeconomics, when one has situations in which either a very large bank fails or many banks fail in a short time period. This event may trigger big losses in real macro variables such as GDP and employment rate and these variables themselves can be interpreted as loss functions. In an economy, given that the dynamics is a consequence of many individual choices, it may be impossible to write down a fully deterministic description for the time evolution. However, it may be sensible to define suitable stochastic processes on the state space of oriented and weighted graphs which are able to phenomenologically reproduce the main statistical features of macroeconomic time evolution. At least, this is what we hope to do in the near future.

## Supporting Information

S1 **R program for Example 1: Rcode_example_1.txt**
(TXT)

S2 **R program for Example 2: Rcode_example_2.txt**
(TXT)
